# Plant‐Derived Monomers for Grey Hair Reversal Through Upregulation of Melanogenesis and Tyrosinase Activity

**DOI:** 10.1111/jcmm.70534

**Published:** 2025-06-03

**Authors:** Chengjie Wei, Xiaomin Hou, Xuelu Jiang, Ming Gao, Yan Gao, Lin Bi, Jisheng Nie, Liangyuan Zhao, Yiwei Shi, Xiaojiang Qin

**Affiliations:** ^1^ Academy of Medical Science Shanxi Medical University Taiyuan Shanxi China; ^2^ Department of Pharmacology Shanxi Medical University Taiyuan Shanxi China; ^3^ Environmental Exposures Vascular Disease Institute Shanxi Medical University Taiyuan Shanxi China; ^4^ Department of Physical Education Shanxi Medical University Taiyuan Shanxi China; ^5^ Department of Foreign Languages Shanxi Medical University Taiyuan Shanxi China; ^6^ School of Public Health Shanxi Medical University Taiyuan Shanxi China; ^7^ Key Laboratory of Coal Environmental Pathogenicity and Prevention Ministry of Education Taiyuan Shanxi China; ^8^ First Hospital of Shanxi Medical University Taiyuan Shanxi China; ^9^ Department of Pulmonary and Critical Care Medicine, NHC Key Laboratory of Pneumoconiosis First Hospital of Shanxi Medical University Taiyuan Shanxi China

**Keywords:** grey hair, melanogenesis, plant‐derived monomers, signalling pathway, tyrosinase activity

## Abstract

Grey hair, a common ageing‐associated phenomenon in humans, is mainly attributed to the damage of melanocytes and the absence of melanin. Grey hair has long been treated with traditional medicine, and new research has shown that various plant‐derived monomers can increase tyrosinase activity and melanogenesis, indicating that they may have therapeutic value in curing grey hair. In this study, we outlined the role of melanin and pigmentation during hair growth and collected various medicinal plant monomers with the potential value of grey hair reversal. Many active ingredients from medicinal plants, such as fraxinol, tribuloside, morin and naringenin, can upregulate melanogenesis and tyrosinase activity through different signalling pathways. Some of them can promote melanosome quantity, maturation and transportation as well. Monomers isolated from medicinal plants may act as stimulators of melanogenesis. Many plant‐derived monomers perform as activators that upregulate melanin synthesis and tyrosinase activity through different signalling pathways. They are of great research value for the treatment of hair greying. Moreover, to further improve experimental effect, safety and reliability, a systematic and comprehensive evaluation system needs to be established in the future before studying their clinical efficacy.

## 1 Introduction

Hair greying, also known as canities, is one of the most common phenotypic and apparent ageing‐associated occurrences in humans [1]. Typically, the onset of hair greying happens at the ages of 34.0 ± 9.6, 43.9 ± 10.3 and 39.0 ± 9.0 in Caucasians, African Americans and Asians, respectively [2]. Scalp hair is vital for sustaining individual self‐confidence and self‐esteem [3]. However, the presence of grey hair may have an impact on personal societal perception, emotional well‐being and psychological state [4]. Numerous endo‐ and exogenous factors are responsible for hair greying; these include internal factors, such as individual genetics, epigenetics and endogenous oxidative stress; external factors, such as ultraviolet (UV) radiation, air pollution, smoking, diet and lifestyle; and the occurrence of certain diseases like vitiligo, which will affect hair follicles (HFs) and cause hair greying [5]. Although the etiopathogenesis of hair greying is exceedingly complex, it is widely thought that the incidence of hair greying is mostly caused by damage to melanocytes located in the hair matrix (HM) near the dermal papillae of HFs, as well as the blockage of melanin synthesis pathways [6, 7].

Numerous studies have demonstrated the beneficial effects of plant‐derived extracts and monomers on hair. Parts of the plant have been shown to have anti‐ageing, anti‐inflammatory and antioxidant qualities [8, 9]. Secondary metabolites found in plants are essential for these characteristics. Based on their chemical structure, these plant monomers are categorised into various chemical groups. Some of them participate in physiological activities via various signalling pathways, including the melanogenic pathway. Microphthalmia‐associated transcription factor (MITF) is the primary regulator of melanogenesis as well as the downstream target. It activates the expression of core genes related to melanogenesis, including genes for tyrosinase (TYR) family enzymes and premelanosome protein, which is the melanosome structural matrix protein [10]. Given that hair dyes cannot entirely solve the problem of grey hair and may cause health hazards [11], it is preferable to treat grey hair with melanogenesis stimulators derived from herbal plants.

In this review, we described the function of melanin and the melanogenesis process during hair growth, especially focusing on the mechanism by which melanogenesis stimulators derived from herbal plants were found to modulate melanin synthesis. Moreover, we pointed out some limitations of the relevant experiment and provided correlative suggestions to improve the reliability and authenticity. We believe that this perspective will inspire the development of therapeutic agents for treating grey hair and improve the accuracy of the experiment in this area.

## 2 Pigmentation During Hair Growth

### 2.1 Biosynthesis of Melanin

Melanin, which is produced by melanocytes, gives human skin, hair and eyes their colour. It serves a variety of physiological purposes, the most significant of which is photoprotecting human skin from UV radiation damage [12, 13, 14]. The biosynthesis and storage of melanin pigments take place in melanosomes, which are special organelles in melanocytes [15]. Numerous studies have documented that a variety of cytokines, including α‐melanocyte‐stimulating hormone (α‐MSH) [16], stem cell factor (SCF) [17], endothelin‐1 (ET‐1) [18], nitric oxide (NO) [19], adrenocorticotropic hormone [20], prostaglandins [21], thymidine dinucleotide [22] and histamine [23], take part in the modulation of melanogenesis. All these factors affect the production of melanin via different signalling pathways by controlling the expression and activation of core pigment‐related genes and proteins, including MITF and TYR family enzymes.

**FIGURE 1:**
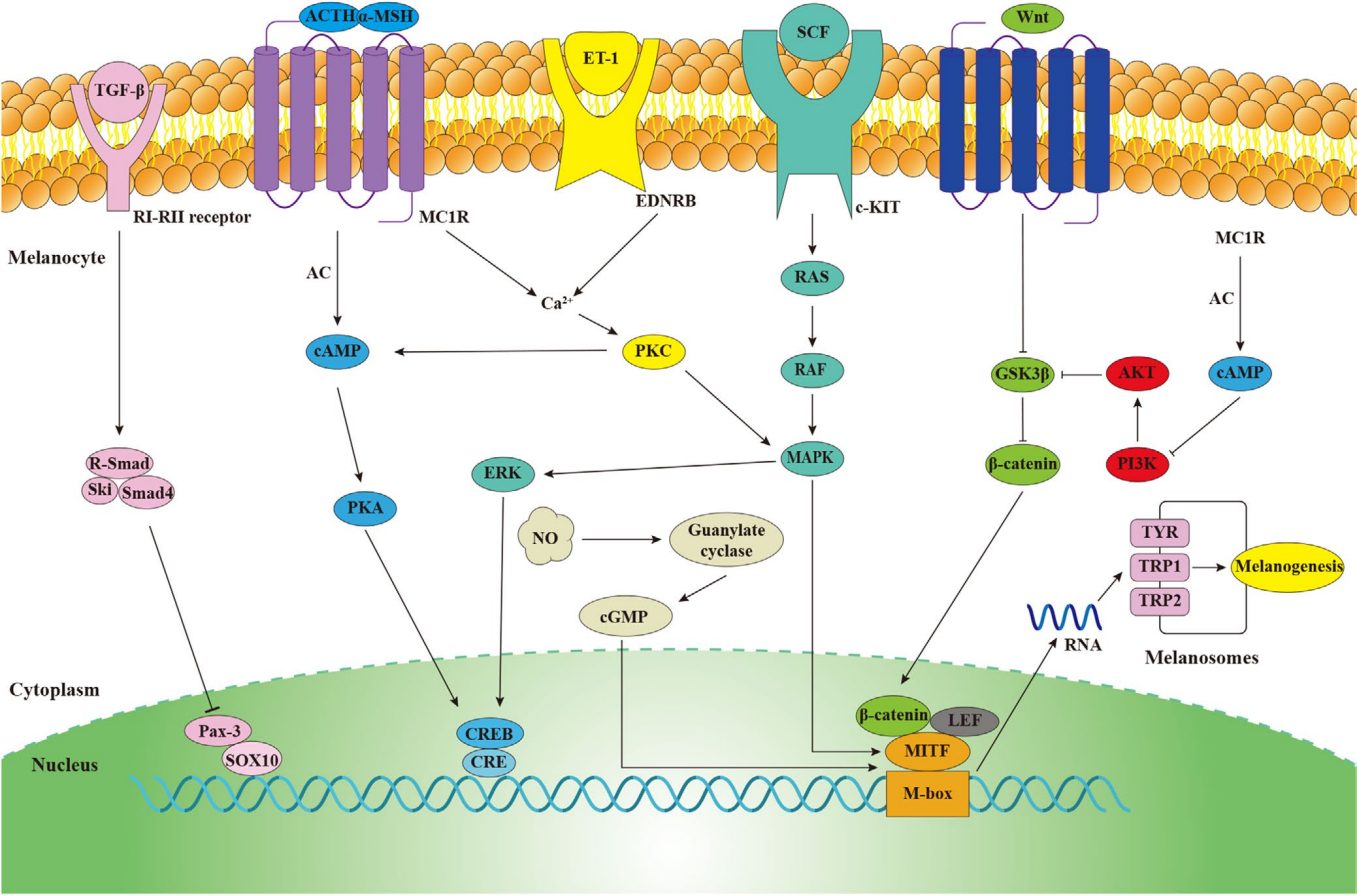
Intracellular signalling pathways in modulation of melanogenesis [24, 25]. A series of intracellular signalling pathways modulate melanogenesis, including TGF‐β, α‐MSH/MC1R, ET‐1/EDNRB, SCF/c‐KIT, Wnt/β‐catenin, PI3K/AKT and NO/cGMP pathways.

A series of intracellular signalling pathways modulate the process of melanogenesis [24, 25]. By binding to the M‐box motif in their promoter regions, all of these signalling pathways modulate MITF to control the expression of key melanogenesis enzymes, such as TYR, tyrosine‐related protein‐1 (TRP‐1) and tyrosine‐related protein‐2 (TRP‐2) (Figure 1) [26, 27]. Specifically, MITF modulates melanocyte differentiation, pigmentation, proliferation and survival [28]. Furthermore, TYR, TRP‐1 and TRP‐2 play primary roles in the enzymatic processes that transform tyrosine into melanin pigments [29].

**FIGURE 2:**
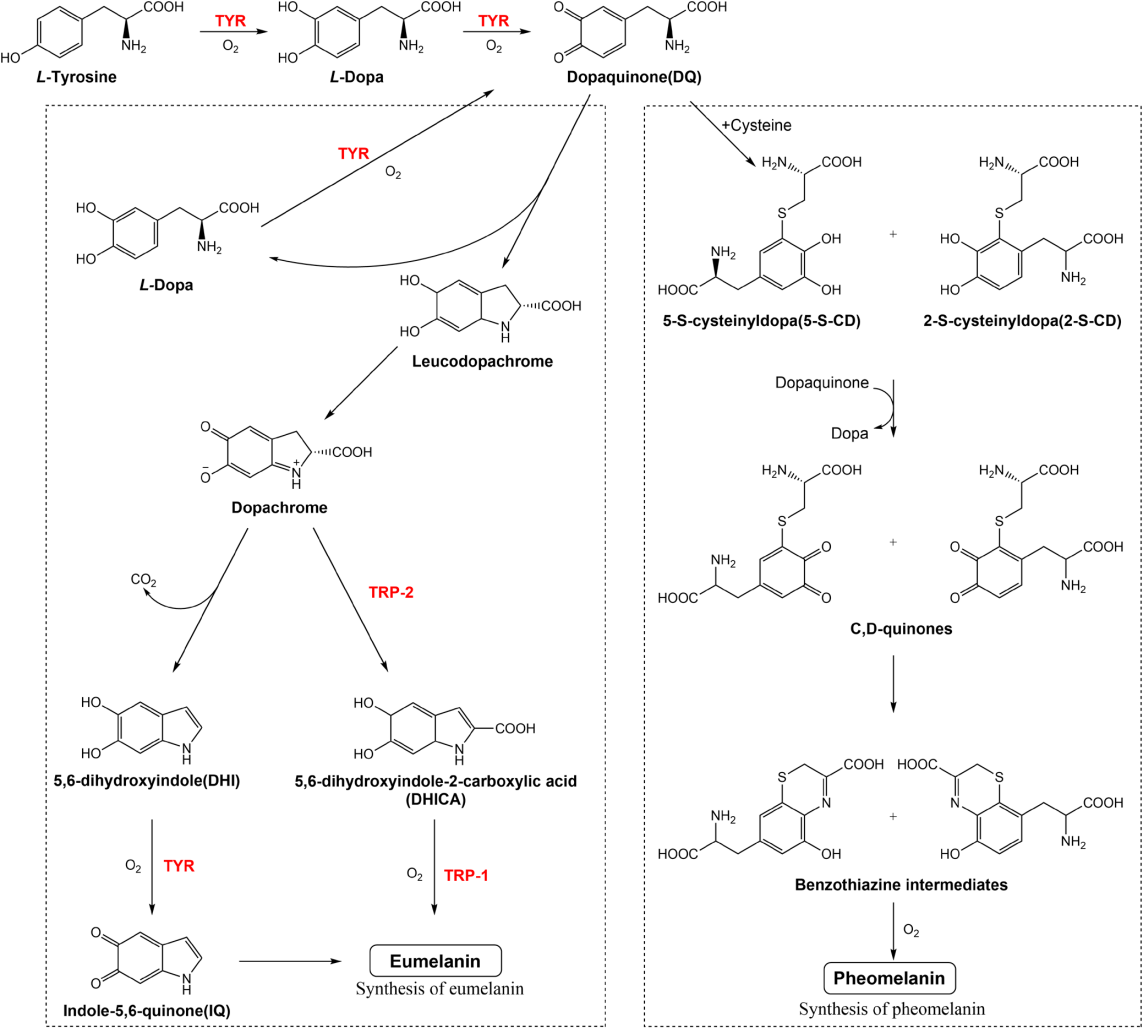
Biosynthesis of eumelanin and pheomelanin [25, 34]. TYR, TRP‐1 and TRP‐2 are involved in the enzyme reactions of melanogenesis; among them, TYR is the exclusive key enzyme.

In humans and other animals, there are two main types of melanin synthesised in melanosomes: Eumelanin, an insoluble polymer that provides brown or black; and pheomelanin, a soluble polymer that provides yellow or red [30]. The colour of hair is directly determined by the quantity and ratio of eumelanin to pheomelanin [31, 32]. Tyrosine, the source of melanin biosynthesis, is commonly regarded as a nonessential amino acid that can be synthesised from phenylalanine [33]. TYR, TRP‐1 and TRP‐2 are engaged in the enzyme processes of melanogenesis; moreover, TYR is the exclusive key enzyme that catalyses the transformation of L‐tyrosine into dopaquinone (Figure 2) [25, 34]. TYR, TRP‐1 and TRP‐2 are known as the enzymes that catalyse the synthesis of eumelanin. After synthesis in the melanosomes, mature melanin is progressively transferred from melanocytes to keratinocytes [35].

### 2.2 Pigmentation During Hair Growth

**FIGURE 3:**
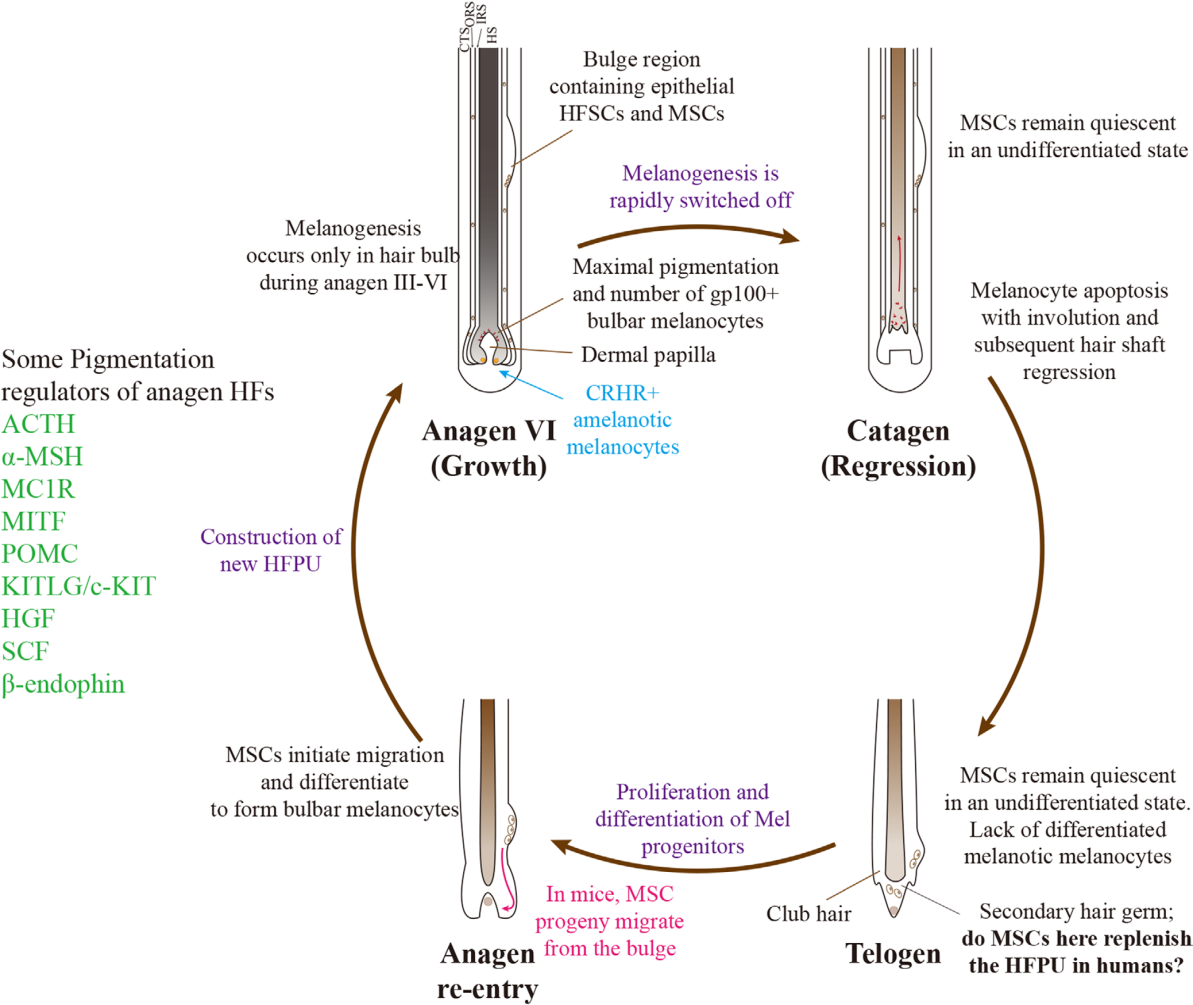
Hair pigmentation during the hair cycle [1, 44].

According to hair growth, the process of the hair cycle can be divided into three major stages: the anagen stage (growth phase), the catagen stage (regressing phase) and the telogen stage (resting phase) [36]. A series of reciprocal interactions between the epithelial cells and dermal papilla cells regulate the formation of HFs and the cycle of hair growth [37, 38]. Melanocyte stem cells (MSCs), which are found in the bulge region of human HFs, are not UVB‐dependent and are strictly cyclical, in contrast to epidermal melanocytes [39]. Melanogenesis occurs solely in the HF pigmentary unit during the anagen III–VI phases of the hair cycle, in membrane‐bound melanosomes, limited to the hair bulb [26, 40, 41]. At anagen VI, with the end of proliferation, melanocytes in the bulb finish differentiating and completely acquire the function of melanogenesis [42]. Hair fibre production and pigmentation are at their peak, and gp100+ and CRHR+ melanocytes are also present in the HM bulb [1]. Finally, the melanosomes are translocated by dendritic and filamentous processes to keratinocytes along the hair shaft [43, 44]. Several factors and signalling pathways, such as MAPK, SCF/c‐KIT, MITF and others, regulate the anagen HF pigmentation process [45]. The process of melanogenesis is swiftly turned off during the anagen–catagen transition stage. Hair shaft regression occurs at catagen because of apoptosis in most keratinocytes in the HM and outer root sheath (ORS), as well as mature melanocytes of the HF pigmentary unit [1, 44, 46]. During telogen, differentiated melanocytes are present in the HM, whereas MSCs remain undifferentiated in the bulge region [1, 47]. In the anagen re‐entry stage, MSCs begin to migrate, proliferate and differentiate down the ORS to produce differentiated melanocytes of the HF pigmentary unit and initiate a new cycle (Figure 3) [1, 44, 48].

### 2.3 Potential Factors for Hair Greying

Above all, a variety of factors, such as melanogenic enzymes, signalling pathways linked to melanogenesis, and the number and survival of melanocytes, can affect melanin production, which in turn affects hair colour. Hence, the mechanism underlying hair greying is intricate. The essential reason for hair greying is the reduction or absence of melanogenesis in HFs [24, 49]. With increasing age, MSCs in the hair bulge decrease or disappear and ectopically differentiated MSCs emerge. This causes a decline in the quantity and viability of MSCs, resulting in insufficient melanocytes for the hair cycle, eventually leading to hair greying [50]. The dysfunction of melanocytes in the hair bulb can temporarily lead to greying as well [51].

Besides, a lack of Bcl‐2 has been shown to cause MSCs to undergo selective apoptosis, which impairs MSC self‐maintenance and causes hair greying [52]. Collagen XVII (Col17a1) is implicated in the regulation of MSC maintenance through hair follicle stem cell (HFSC)–generated TGF‐β signalling, and its absence contributes to premature hair greying and hair loss [53]. The expression of Col17a1 is found in HFSCs but not in MSCs. Fortunately, hair greying may be reversed by reactivating the remaining MSCs or amelanogenic melanocytes [51].

It was reported that hair greying is related to active hair growth [54]. Van Neste [55] discovered that the growth of non‐pigmented HFs was more rapid than pigmented HFs. However, it seems to be the consequence of the lack of melanin in keratinocytes rather than the cause of greying hair. Thus, more evidence should be explored to confirm the specific relationship between melanin and hair growth.

## 3 Plant‐Derived Monomers for Promoting Melanogenesis

Many plant‐derived compounds have shown the ability to promote melanogenesis and TYR activity. The pure substances derived from plants that have the capacity to stimulate melanogenesis can be categorised into a number of chemical classes based on their chemical structures, including coumarins, flavonoids, terpenoids, polyphenols and others.

### 3.1 Coumarins

Coumarin is widely distributed in different parts of plants and has the highest concentration in fruits, seeds, roots and leaves [56, 57]. The character of coumarin is a benzene ring fused with a pyrone ring. Several studies have discovered that coumarins have the ability of melanogenesis, which may apply to the treatment of hair greying.

**FIGURE 4:**
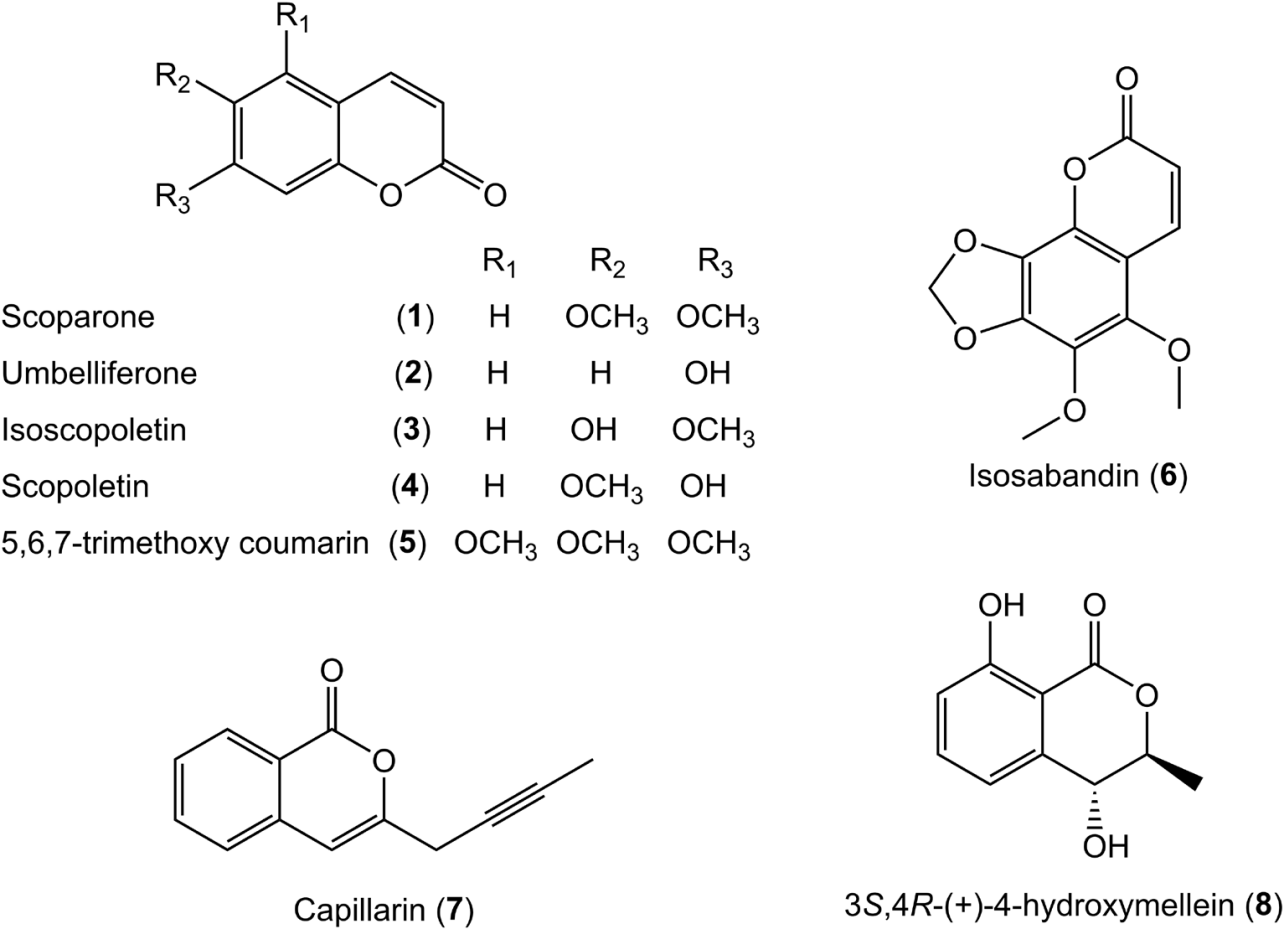
Structures of coumarins from aerial parts of *Artemisia scoparia* Waldst. et Kit.

Recently, 16 compounds with 8 known coumarins (**1**–**8**) (Figure 4) were isolated and identified from the aerial parts of *Artemisia scoparia* Waldst. et Kit., an herbal plant traditionally applied for fever, inflammation, jaundice and infection [58]. These compounds were evaluated for their cytotoxicity, anti‐inflammatory and anti‐vitiligo activities. All of them (1 μM) showed varying degrees of promoting melanogenesis and TYR activity in B16 cells [59]. However, more research on the possible mechanism is required in the future.

**FIGURE 5:**
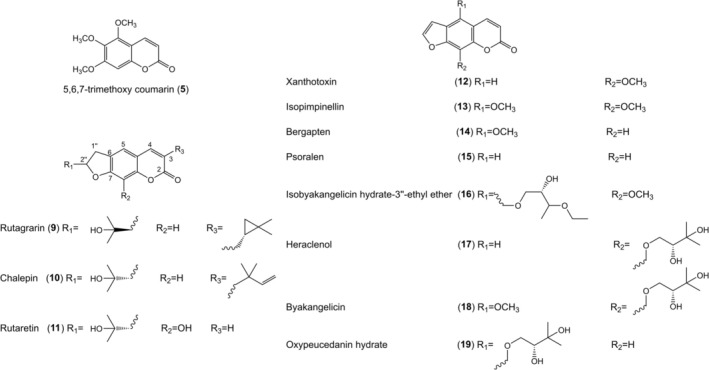
Structures of coumarins from aerial parts of *Ruta graveolens* L.

Ainiwaer et al. [60] isolated and identified 15 compounds with 12 coumarins (**5**, **9–19**) (Figure 5) from the aerial parts of *Ruta graveolens* L. with anti‐vitiligo effects [61], which were assessed for their activities on skin cell melanogenic biology in B16 melanocytes. As a result, compounds **5**, **12**, **13**, **14** and **15** increased melanin contents and improved TYR activity in B16 melanoma cells in a concentration‐dependent manner. These ingredients' activation ability was comparable to that of 8‐methoxypsoralen at 50 μM. Moreover, compared with the positive control, isopimpinellin (compound **13**) (50 μM) increased melanin synthesis by more than 50%. Further research indicated that isopimpinellin enhances melanogenesis by activating the AKT/GSK3β/β‐catenin pathway to increase the expression level of phosphorylated AKT, GSK‐3β, β‐catenin‐ser 33, 37, 41 and total β‐catenin and decrease the expression level of p‐β‐catenin‐ser 675 to upregulate MITF and TYR family genes in a dose‐dependent manner.

Other research discovered that scopoletin (**4**), a derivative coumarin that can be isolated from *Cirsium setidens* Nakai (Compositae), a medicinal plant with antioxidant, anti‐inflammatory and anti‐tumour effects [62], can notably improve the content of melanin by increasing levels of phosphorylated CREB to strongly induce the expression levels of MITF, TYR and TRP‐1 in a dose‐ and time‐dependent manner in B16F10 cells. It was reported that the potential mechanism of scopoletin induces melanogenesis via the cAMP/PKA signalling pathway and partial p38 MAPK activation [63, 64].

Fraxinol (**20**), a natural coumarin extracted from *Fraxinus* plants with several pharmacological properties like antioxidant, immunomodulatory and anti‐inflammatory [65], can effectively stimulate melanogenesis in B16F10 cells in a concentration‐dependent manner without cytotoxicity (100 μM). By phosphorylating CREB, fraxinol has been shown to boost MITF expression through the PKA‐dependent CREB/MITF/TRP‐1 signalling pathway, therefore increasing the mRNA expression of melanogenic enzymes, such as TYR, TRP‐1 and TRP‐2, as well as the expression at the mRNA and protein levels of MITF [66].

Lee et al. [67] reported that 7,8‐dimethoxycoumarin (DMC, C_11_H_10_O_4_) (**21**), a natural coumarin existing in several herbal plants, could significantly increase the melanin content and TYR activity in B16F10 melanoma cells in a concentration‐dependent manner without cytotoxicity (0.4 mM). The research indicated that DMC increases the expression of TYR, TRP‐1, TRP‐2 and MITF to activate the production of melanin by interfering with ERK phosphorylation in the MAPK signalling pathway and increasing AKT phosphorylation in the AKT signalling pathway. By phosphorylating GSK‐3β in ser21/9, the improvement in AKT phosphorylation causes β‐catenin to accumulate. When β‐catenin accumulates in the cytoplasm, it translocates to the nucleus and promotes MITF expression. However, the interference of ERK phosphorylation in the MAPKs pathway may reduce the production of melanin.

8‐Methoxycoumarin (**22**) extracted and purified from *R. graveolens* L. can increase the melanin content of the B16F10 murine cells without cytotoxicity (400 μM). It was suggested that 8‐methoxycoumarin can promote the phosphorylation of AKT and MAPK, which in turn upregulates the expression of TYR, TRP‐1 and TRP‐2 to stimulate melanogenesis via the MAPK signalling pathway [68].

### 3.2 Flavonoids

Widely distributed in plants with diverse health benefits, flavonoids are the most abundant type of natural polyphenols [69, 70]. The basic structure of flavonoids contains two parts: benzopyran (A and C ring) and phenyl (B ring) with carbon structure C6–C3–C6. Certain flavonoids exhibit anti‐vitiligo properties, suggesting their potential application in the treatment of hair greying.

**FIGURE 6:**
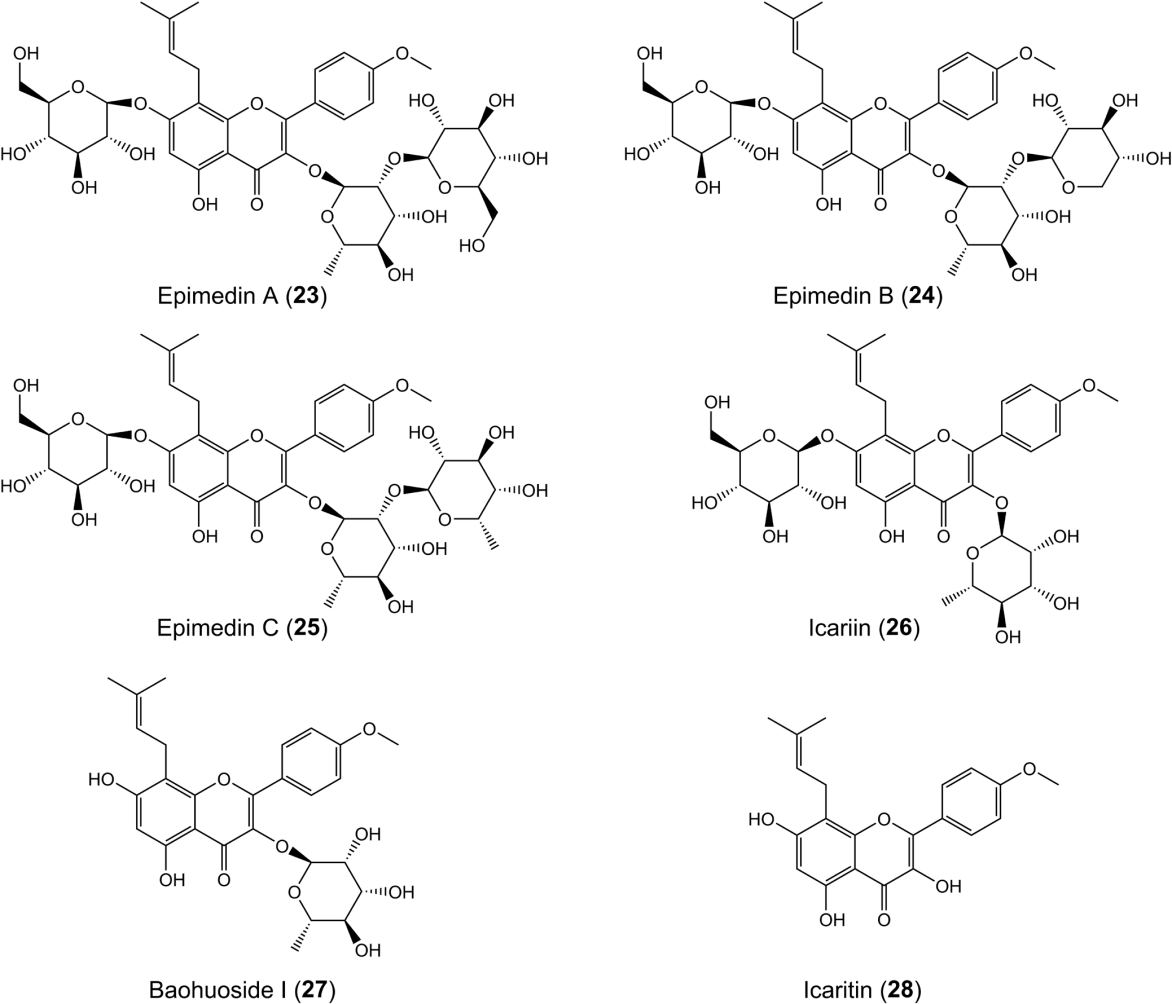
Structures of flavonoids from *Epimedium brevicornum* Maxim.

Hong et al. [71] isolated and identified 6 flavonoids (**23–28**) (Figure 6) from *Epimedium brevicornum* Maxim., a traditional medicine widely utilised in China for sexual dysfunction and osteoporosis treatment, which was evaluated for the pigmentation effects on melanin synthesis and melanosome biogenesis/transfer. Consequently, epimedin A, epimedin B and epimedin C were shown to promote melanogenesis and TYR activation in varying degrees. At the same concentration, epimedin B exhibited the best results in terms of TYR and melanogenesis. Their further research showed that epimedin B (**24**) increased the number of melanosomes and their maturation stage, as well as the expression of TYRs, in a dose‐ and time‐dependent manner by controlling the p‐AKT‐mediated GSK3β/β‐catenin, p‐p70S6K, p38/MAPK and ERK/MAPK pathways. Additionally, epimedin B increased the activity and stability of TYRs by preventing the formation of aberrant TYR and TRP‐1, retaining them in the endoplasmic reticulum and preventing ubiquitin‐proteasome degradation [72].

Previous studies have shown that tribuloside (**29**), a natural flavonoid isolated from the water‐based extract from traditional Chinese medicine *Tribulus terrestris* L., whose fruits have been utilised in China to treat migraines, dizziness and vitiligo, enhances melanogenesis in mouse hair follicle melanocytes by elevating the expression levels of α‐MSH and MC1R to stimulate melanogenesis. Recently, Cao et al. [73] conducted an in‐depth study of the mechanism of tribuloside on pigmentation and discovered that tribuloside showed a notable impact on the production of melanin in melanocytes, zebrafish and human skin samples without toxic effects on cells (40 μM). Research suggested that tribuloside enhances the melanocyte dendricity and melanosome transport by upregulating Rab 27a, Rab 17 and Cdc 42. Furthermore, tribuloside enhances the expression of p‐PKA cat, p‐CREB and p‐p38 and inhibits PDE to increase the intracellular level of cAMP to induce MITF expression for which tribuloside acts on the PDE/cAMP/PKA pathway to enhance melanogenesis, melanocyte dendricity and melanosome transport.

Morin (**30**), a well known flavonoid naturally found in various mulberries and herbs, has found that it exhibited no cytotoxicity in the concentration range of 5–100 μM. In B16F10 mouse melanoma cells, morin enhanced the phosphorylation of ERK and p38 to improve the expression of TRP‐1, TRP‐2 and MITF, which regulates the synthesis of melanin [74]. After 12 h, the level of phosphorylated ERK decreased. The MAPK pathway is activated or regulated in numerous phases, with ERK and p38 being the main kinases. The results indicated that morin stimulates the synthesis of melanin via the MAPK signalling pathway.

Silibinin (**31**), a flavonolignan isolated from milk thistle ( *Silybum marianum* ), which is usually used to manage liver diseases, has shown that it markedly enhanced the content of melanin in murine B16‐F1 and human HMV‐II melanoma cells. Silibinin motivates the activity of intracellular TYR and the expression of TYR, TRP‐1, TRP‐2 and MITF to improve melanogenesis through PKA and p38 MAPK signalling pathways, leading to the phosphorylation of CREB and the expression of MITF [75].

Kaempferol (**32**), a representative flavonoid constituent of *Sanguisorba officinalis* , is known as a traditional herb for the treatment of pigment loss [76]. Melanocytes treated with kaempferol showed perimembranous accumulation of HMB45‐positive melanosomes as well as enhanced expression of Rab27A, RhoA and Cdc42, which improved melanosome transport to perimembranous actin filaments to promote melanogenesis and melanocyte growth. In addition, kaempferol increased the phosphorylation of P38/ERK MAPK while downregulating the expression of p‐PI3K, p‐AKT and pP70s6K to enhance melanogenesis. Above all, kaempferol modulated melanocytes' dendritic growth, melanosome quantity, maturation, transport and melanogenesis via the P38/ERK MAPK and PI3K/AKT signalling pathways [77].

The molecular mechanism of melanogenesis was studied in relation to liquiritin and liquiritigenin (**33** and **34**), the main flavonoids found in licorice root (*Glycyrrhiza* spp.), an herb plant with a variety of medicinal properties, including anti‐inflammatory, antiallergenic and liver function improvement [78]. It was shown that these compounds increased the expression of melanogenic enzymes by upregulating the phosphorylation level of p38 and ERK, which in turn induced melanin synthesis and intracellular TYR activity. This indicates that liquiritin and liquiritigenin regulate melanogenesis through the p38 and ERK pathways [78].

Naringenin (**35**), a flavanone that mainly exists in the *Citrus* species, has been found to enhance the content of melanin in murine B16F10 melanoma cells [79]. It was indicated that naringenin induces phosphorylated AKT or GSK3β to promote the expression of TYR, MITF and β‐catenin in a concentration‐dependent manner. The results showed that naringenin improves melanin synthesis through motivating the PI3K/AKT or Wnt/β‐catenin signalling pathways [80].

### 3.3 Terpenoids

As the largest and most diverse class of chemicals among the myriad natural plant secondary metabolites, terpenoids can be found in bushes, grass, trees and even in milk [81, 82]. With isoprene as the basic structural unit, terpenoids can be mainly classified into monoterpenoids, diterpenoids, triterpenoids, sesquiterpenoids and polyterpenoids [81]. Some of them have been found to upregulate melanogenesis and activate melanosome maturation, transport and dendritic growth in melanocytes.

Paeoniflorin (**36**) is the major monoterpenoid found in the root of *Paeonia lactiflora* Pall., a traditional Chinese medicine with antioxidant stress effects [83]. Paeoniflorin was discovered to notably increase intracellular TYR activity and melanin content at a concentration of 10 μg/mL. Furthermore, it was demonstrated that paeoniflorin can induce the phosphorylation of CREB and ERK to upregulate the levels of MITF and TRP‐1 and enhance the synthesis of melanin, suggesting that paeoniflorin promotes melanogenesis through the ERK/CREB signalling pathway [84].

Lan et al. [85] reported geniposide (**37**), an iridoid glycoside isolated from the fruit of *Gardenia jasminoides* Ellis, which is known as a Chinese traditional herbal for its anti‐vitiligo effect and explored the mechanism for enhancing melanogenesis in 0.1 μM norepinephrine‐exposed normal human epidermal melanocytes. These findings demonstrated that geniposide increased keratinocytes' phosphorylation of SCF, which activated c‐Kit carried by melanocytes in turn to successfully promote melanogenesis. This suggests that geniposide stimulates melanogenesis through the SCF/c‐Kit pathway.

Lupenone (**38**) is an active component isolated from the leaves of *Erica multiflora* L., a plant with anti‐inflammatory and hypoglycaemic activity [86]. It has been demonstrated that lupenone does not affect TYR activity; however, it may increase TYR transcription and translation levels. It was noticeable that lupenone promotes melanogenesis by stimulating the expression of the TYR enzyme through MAPK‐phosphorylated ERK1/2 phosphorylation inhibition [87].

### 3.4 Polyphenols

Polyphenols represent a large family of secondary metabolites generated by plants [88]. Polyphenols originate from phenylalanine or a close precursor, shikimic acid, and the basic unit of polyphenols is the phenol ring [89]. Numerous researches have demonstrated the preventive function of polyphenols in skin care, with some even finding that they increase melanogenesis.

A common phenol derivative found in plants, rosmarinic acid (**39**), was isolated and purified from the well‐known vegetable species *Salvia officinalis* L., which is used to season food. It exhibited a dual behaviour on melanogenesis: At low concentrations, it stimulated the production of melanin and TYR activity, whereas at higher concentrations, it inhibited them [90]. It was reported that rosmarinic acid activated CREB to induce melanogenesis through the PAK signalling pathway [91]. Rosmarinic acid alone may not be the only compound that promotes melanogenesis; some extracts, like the polyphenolic extract of lemon balm ( *Melissa officinalis* L.) and extracts of *S. officinalis* L., have demonstrated a greater capacity for melanogenesis [90, 92].

The natural polyphenol 1,5‐dicaffeoylquinic acid (1,5 ‐ diCQA) (**40**) was extracted from the seeds of *Vernonia anthelmintica* (L.) Willd., an herb plant with several pharmacological properties, including anti‐inflammatory, antibacterial, antioxidant and anti‐vitiligo [93]. 1,5 ‐ DiCQA promotes melanin through two pathways: on the one hand, it increases the levels of p38 MAPK and ERK MAPK phosphorylation to activate MITF; on the other hand, it increases the cAMP content, which activates PKA that subsequently phosphorylates CREB to activate MITF [94]. This indicates that 1,5 ‐ diCQA promotes melanin content through the MAPK and cAMP/PKA pathways in B16 cells.

### 3.5 Other Compounds

Theophylline (**41**), a purine alkaloid and the main ingredient in tea plants, has been found to improve the protein expression levels of MITF, TYR, TRP‐1 and β‐catenin and promote the phosphorylation of ERK and GSK3β to increase melanin content in B16F10 murine melanoma cells. Above all, theophylline increases melanin production by activating the MEK1/2 and Wnt/β‐catenin signalling pathways [95].

An ingredient named isofraxidin 7‐*O*‐(6′‐*O*‐p‐Coumaroyl)‐β‐glucopyranoside (**42**) is extracted from *Artemisia capillaris* Thunberg, a well‐known herbal plant that has been used to treat malaria, inflammation and cancer [96]. It was suggested that this compound can enhance the expression of TYR and MITF in melanocytes in a dose‐dependent manner. Furthermore, in the zebrafish model, it could upregulate melanin production and TYR activity without toxicity [97]. However, more research is required to determine the specific mechanism of the upregulated melanin content.

*Ruta graveolens* L. produced a novel alkaloid (**43**) that was found to increase the content of melanin. Based on bioactivity studies, kokusaginine may regulate interleukin‐6 and endoplasmic reticulum stress to protect PIG3V melanocytes from oxidative damage brought on by 4‐tert‐butylphenol [98]. Additionally, it had an antioxidant impact by suppressing the expression of Bip, IRE1, p‐IRE1 and XBP‐1 proteins. It is worthwhile to conduct additional research to better understand how this chemical works to treat vitiligo.

2,3,5,4′‐Tetrahydroxystilbene‐2‐O‐β‐D‐glucoside (THSG) (**44**), a water‐soluble glycoside isolated from the dried tuber root of *Polygonum multiflorum* Thunb., a well‐known traditional Chinese herb for the effect of blackening hair. By triggering CREB activation and p38 MAPK phosphorylation, it has been demonstrated that THSG increases melanin production and TYR activity in a concentration‐dependent and time‐dependent manner [99]. These findings suggest that THSG induces melanogenesis through cAMP/PKA and MAPK signalling pathways.

Epigallocatechin‐3‐gallate (EGCG) (**45**), a main component in green tea, was evaluated for its efficiency in treating vitiligo induced by monobenzone in mice. It was indicated that EGCG could notably reduce histopathologic changes in the skin by downregulating excessive inflammatory responses, especially infiltration of CD8^+^ T cells and significantly inhibiting the inflammatory mediators' levels compared with the model group [100]. These consequences indicated that EGCG may be a potential agent for the treatment of greying hair.

**FIGURE 7:**
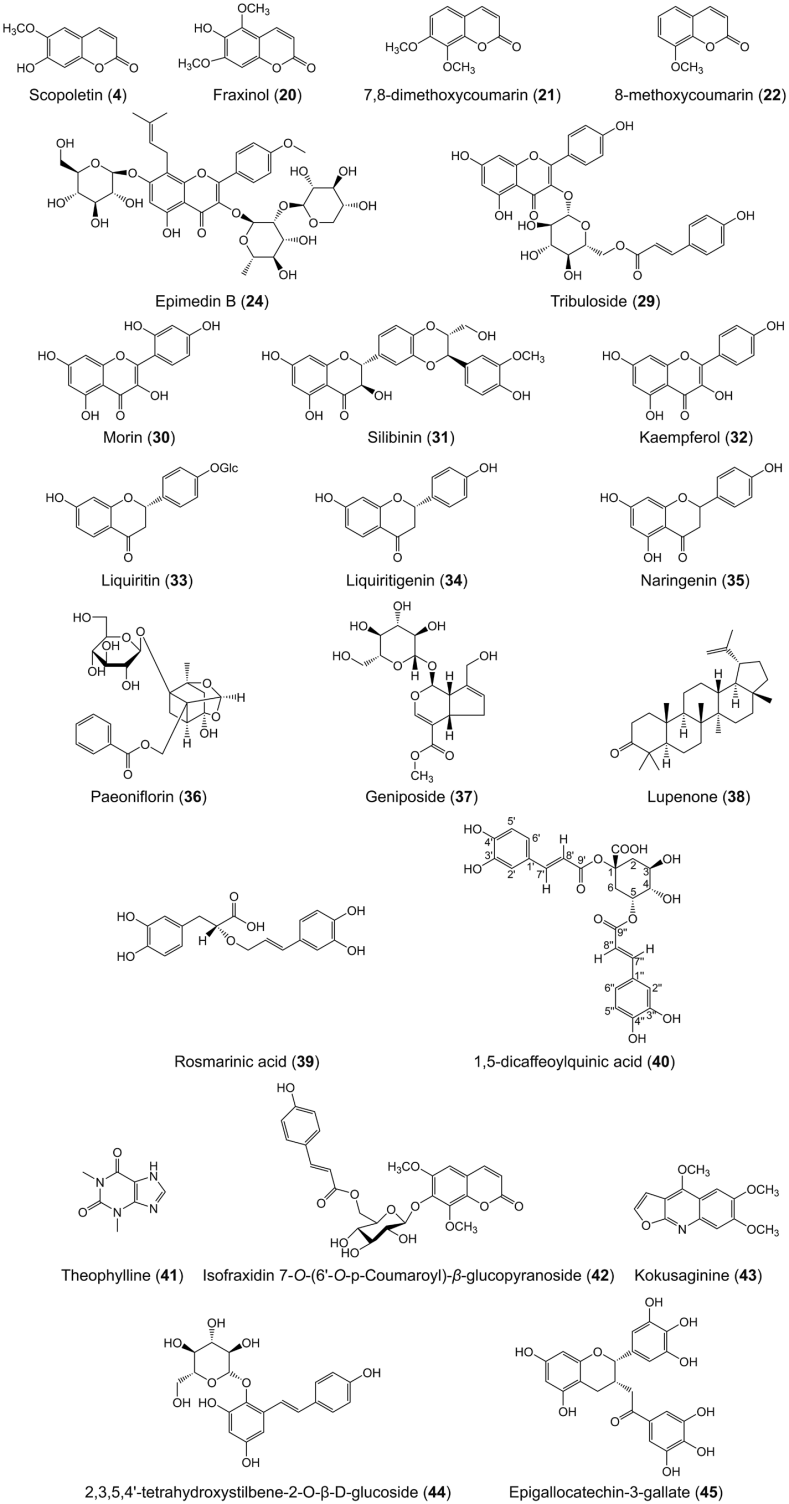
Chemical structures of monomers with stimulator effects on melanogenesis from herbal plants.

The molecular structures of the monomers isolated from various medicinal plants that can stimulate melanogenesis and improve TYR activity are shown below (Figure 7).

The effects of active monomers isolated from plants that can induce melanogenesis are summarised in Table 1. These monomers might be employed as agents to reverse grey hair.

**TABLE 1:** Effects of active monomers isolated from herbal plants on melanogenesis.

Categories	Monomers	Herbal plants	Extract parts	Types of cells/doses	Animal experiments/doses	Upregulated items	Related signalling pathways	References
Coumarins	Scopoletin	*Cirsium setidens* Nakai (Compositae)	Aerial	B16F10/0.1, 1, 10, 20, 40 and 50 (μM)	—	Melanin, tyrosinase activity, MITF expression and CREB phosphorylation	cAMP/PKA pathway and partially p38 MAPK activation	[63, 64]
	Fraxinol	*Fraxinus*	—	B16F10/0, 20, 40, 60, 80 and 100 (μM)	—	Melanin, tyrosinase activity, MITF expression and CREB phosphorylation	PKA/CREB/MITF/TRP‐1 pathway	[66]
	DMC	*Daphne koreana*; *Astianthus viminalis*; *Zanthoxylum leprieurii* and *Citrus decumana*	—	B16F10/0.05, 0.1, 0.2 and 0.4 (mM)	—	Melanin, tyrosine activity, MITF expression and AKT phosphorylation	AKT pathway and MAPK pathway	[67]
	8‐Methoxycoumarin	*R. graveolens* L.	—	B16F10/0, 50, 100, 200 and 400 (μM)	—	Melanin, tyrosine activity and MITF expression	MAPK pathway	[68]
Flavonoids	Epimedin B	*Epimedium brevicornum* Maxim.	Leaves	B16F10/0, 25, 50 and 100 (μM) MNT‐1/0, 25, 50 and 100 (μM)	Zebrafish/0.5, 1 and 5 (mM) Male model mice/0.1%, 4%	Melanin, tyrosinase activity, expression and stability	p‐AKT‐mediated GSK3β/β‐catenin, p‐p70S6K, P38/MAPK and ERK/MAPK pathways	[71, 72]
	Tribuloside	*Tribulus terrestris* L.	—	HEMC/0, 5, 10 and 20 (μM)	Zebrafish/0, 5, 10 and 20 (μM)	Melanin, tyrosinase activity, MITF expression, melanocytes' dendric growth and melanosome transport	PDE/cAMP/PKA pathway	[73]
	Morin	*Morus alba* L.	Woods	B16F10/0, 25, 50 and 100 (μM)	—	Melanin, tyrosinase activity, MITF expression	MAPK pathway	[74]
	Silibinin	*Silybum marianum*	Seeds	B16F1/0, 5, 10 and 20 (μM) HMV‐II/0, 5, 10 and 20 (μM)	—	Melanin, tyrosine activity, MITF expression, CREB, PKA and p38 MAPK phosphorylation	PKA/CREB pathway and p38 MAPK pathway	[75]

	Kaempferol	*Sanguisorba officinalis* L.	Root	Melan‐A/15 μM B16F10/5–25 (μM) SK‐MEL‐28/15 μM	Zebrafish/15 μM	Melanin, melanocytes' dendritic growth, MITF expression, melanosome quantity, maturation and transport	P38/ERK MAPK pathway and PI3K/AKT pathway	[77]
	Liquiritin	*Glycyrrhiza* spp.	Root	B16F1/0, 12.5, 25 and 50 (μM) HMV‐II/0, 12.5, 25 and 50 (μM)	—	Melanin, tyrosine activity, MITF expression, CREB, p38 and ERK phosphorylation	p38 and PKA pathways	[78]
	Liquiritigenin	*Glycyrrhiza* spp.	Root	B16F1/0, 12.5, 25 and 50 (μM) HMV‐II/0, 12.5, 25 and 50 (μM)	—	Melanin, tyrosinase activity, MITF expression, CREB, p38 and ERK phosphorylation	p38 and PKA pathways	[78]
Naringenin	*Citrus*	Fruit	B16F10/0, 3, 10, 30 and 50 (μM)	—	Melanin, tyrosinase activity, MITF expression, β‐catenin accumulation, GSK3β phosphorylation and PI3K activity	Wnt/β‐catenin pathway	[79, 80]
Terpenoids	Paeoniflorin	*Paeonia lactiflora* Pall.	Root	HMC/0, 2.5, 5, 10, 20, 50, 100, 200, 500 and 1000 (μg/mL)	Vitiligo model female C57BL/6 mice/60 (mg/kg)	Melanin, tyrosinase activity, MITF expression, CREB and ERK phosphorylation	ERK/CREB pathway	[84]
	Geniposide	*Gardenia jasminoides* Ellis	Fruit	Norepinephrine‐exposed HEMC/0, 1, 10 and 100 (μM)	—	Melanin, c‐Kit expression	SCF/c‐Kit pathway	[85]
	Lupenone	*Erica multiflora* L.	Leaves	B16/0.1, 0.5 and 1.0 (μM)	—	Melanin, MITF expression	p‐ERK1/2 MAPK pathway	[87]
Polyphenols	Rosmarinic acid	*Salvia officinalis* L.	Leaves	B16F10/0, 0.001, 0.01, 0.1, 1, 10, 100 and 1000 (μM)	—	Melanin, tyrosinase activity	PKA pathway	[90, 91]
	1,5‐Dicaffeoylquinic acid	*Vernonia anthelmintica* (L.) Willd.	Seeds	B16/0, 5, 25, 50, 100, 200 and 400 (μM)	—	Melanin, tyrosinase activity, MITF expression, p38 MAPK, ERK and CREB phosphorylation	MAPK pathway and cAMP/PKA pathway	[94]
Others	Theophylline	Tea	Leaves	B16F10/0, 100, 250 and 500 (μM)	—	Melanin, MITF expression, ERK and GSK3β phosphorylation	MEK1/2 pathway and Wnt/β‐catenin pathway	[95]
	Isofraxidin 7‐*O*‐(6′‐*O‐*p‐Coumaroyl)‐β‐glucopyranoside	*Artemisia capillaris* Thunberg.	—	B16F10/25 (μM)	Zebrafish and embryos/12.5, 25 (μM)	Melanin, tyrosinase activity and MITF expression	—	[97]
	Kokusaginine	*Ruta graveolens* L.	Aerial	B16/0, 1, 10, 50, 100 and 200 (μM) PIG3V/0, 1, 10, 50, 100 and 200 (μM)	—	Melanin, managed ER stress and ameliorate vitiligo exacerbation	—	[98]
	THSG	*Polygonum multiflorum* Thunb.	Root	B16/1, 2, 5 and 10 (μg/mL)	—	Melanin, tyrosinase activity, MITF expression, CREB activation and p38 MAPK phosphorylation	MAPK pathway	[99]
	EGCG	Green tea	—	—	Vitiligo model female C57BL/6 mice/2%, 5% and 10%	Delayed depigmentation time, reduced the prevalence of depigmentation, decreased depigmentation area and decreased perilesional accumulation of CD8^+^ T cells	—	[100]

## 4 Limits and Existing Issues

Many plant‐derived monomers have been proven to regulate melanogenesis and TYR activity through various signalling pathways. Some of them can modulate melanosome maturation and transport. However, some limitations and concerns should be addressed in future research [76].

### 4.1 Sources of Melanogenesis Stimulators

In the current review, several investigations on a single active ingredient demonstrate that pharmaceuticals are made from bought chemicals instead of plant extracts. In most cases, the purity of the active compounds that researchers extract is not indicated. Furthermore, the provenance of plants has a significant impact on the variations in the active ingredients found in medicinal herbs. It is noteworthy to label the origins of medicinal plants and carry out comparative studies on the variations in composition and effectiveness of medicinal plants from various geographical areas.

Furthermore, variations in the content of certain monomers may result from various extraction techniques and environmental factors. It is important to conduct more research to determine the best techniques for extracting and purifying certain monomers.

### 4.2 Pharmacological Process of Melanogenesis Stimulators During Melanogenesis

There are still several pharmacological effects of monomers and processes on melanogenesis that require improvement and clinical data validation. Some research only demonstrated the role of monomers to promote melanogenesis, but the specific mechanism and upstream signalling pathways are still unknown. Additional research is necessary to determine the precise stimulating effect of melanin production. The mechanism of plant ingredients' effects on melanogenesis is the primary focus of many investigations; nevertheless, the way compounds change structure during metabolism offers important information for future research and the creation of more potent components.

The majority of studies focus on the melanogenic activity of a single component while neglecting the potential synergistic effects of other components. Certain monomers have shown dual activity on melanogenesis; however, such components in the plant extracts exhibit concentration‐dependent behaviour on melanin production. Future studies on the possible synergistic mechanisms of various plant monomers are worthwhile.

### 4.3 Experiments of Plant Monomers on Melanogenesis

The B16 cell line, a melanoma cell originated from C57 mice that can produce melanin in vitro, is an excellent resource for cell research on melanogenesis. However, the distinctions between mouse and human cells indicate that data from experiments with the B16 cell line cannot be fully extrapolated to human cells. The A375 cell line, a human melanoma cell that generates little melanin in vitro, is unsuitable for researching the effects of drugs on melanogenesis. Thus, we should utilise human primary melanocytes or other human cell lines that can produce melanin to undertake follow‐up studies on medicinal plants for the treatment of pigmentary diseases.

Current animal models of grey hair are unable to sufficiently explain the mechanism of human grey hair, and chemically generated models cannot directly reflect the pathophysiology of grey hair. Future studies on grey hair in animal models are valuable.

It is necessary to thoroughly examine allergies and other plant effects in addition to cytotoxicity and positive effect tests. Dosage, toxicity and administration technique should all be carefully considered during studies using cell and animal models. If the administration method is changed from external to internal, in addition to the treatment effect, the toxicity study and safety evaluation should be given careful consideration.

## 5 Conclusion and Prospects

Plant secondary metabolites are abundant sources for treating pigmentary diseases, and many phytochemicals have been proposed as potential melanogenesis stimulators for the treatment of grey hair. Many of them have been shown to activate melanin synthesis and activity via different signalling pathways.

As previously mentioned, we discovered that a variety of compounds increase TYR activity and melanogenesis through the MAPK signalling pathway. Furthermore, certain flavonoids have the ability to influence melanosome quantity, maturation and transport, which in turn affects melanogenesis, in addition to increasing TYR activity. According to these studies, there is a great deal of promise in using flavonoids to treat grey hair.

Although melanogenesis stimulators have been shown to be beneficial in promoting melanin content, tyrosine activity and treating vitiligo, many of them have yet to be employed in experiments to improve grey hair. Furthermore, the experimental effect, safety and reliability of the present investigation are limited. Therefore, a systematic and comprehensive evaluation system needs to be established for future research. Future research on plant‐derived monomers and medicinal plants for the treatment of hair greying will benefit from the findings given in this work.

## Author Contributions

**Chengjie Wei:** conceptualization (equal), methodology (equal), writing – original draft (equal). **Xiaomin Hou:** formal analysis (equal), methodology (equal). **Xuelu Jiang:** formal analysis (equal), methodology (equal). **Ming Gao:** formal analysis (equal). **Yan Gao:** writing – review and editing (equal). **Lin Bi:** data curation (equal), methodology (equal). **Jisheng Nie:** investigation (equal), validation (equal). **Liangyuan Zhao:** formal analysis (equal), software (equal). **Yiwei Shi:** validation (equal), visualization (equal). **Xiaojiang Qin:** conceptualization (equal), formal analysis (equal), funding acquisition (equal), methodology (equal), project administration (equal), supervision (equal), writing – review and editing (equal).

## Ethics Statement

The authors have nothing to report.

## Consent

The authors have nothing to report.

## Conflicts of Interest

The authors declare no conflicts of interest.

## Data Availability

All data generated or analysed during this study are included in this published article.
